# Analyzing non-sentinel axillary metastases in patients with T3–T4 cN0 early breast cancer and tumor-involved sentinel lymph nodes undergoing breast-conserving therapy or mastectomy

**DOI:** 10.1007/s10549-020-05876-z

**Published:** 2020-08-20

**Authors:** Fabian Riedel, Joerg Heil, Manuel Feisst, Mareike Moderow, Alexandra von Au, Christoph Domschke, Laura Michel, Benedikt Schaefgen, Michael Golatta, André Hennigs

**Affiliations:** 1 Department of Gynecology and Obstetrics, Heidelberg University Hospital, Im Neuenheimer Feld 440, 69120 Heidelberg, Germany; 2 Institute of Medical Biometry and Informatics, Heidelberg University Hospital, Im Neuenheimer Feld 130.3, 69120 Heidelberg, Germany; 3 West German Breast Center GmbH, Bahlenstr. 180, 40589 Düsseldorf, Germany

**Keywords:** Breast cancer, Axillary lymph node dissection, Sentinel lymph node dissection, Non-sentinel axillary metastases, ACOSOG Z0011 trial

## Abstract

**Purpose:**

In the ACOSOG Z0011 trial, completing axillary lymph node dissection (cALND) did not benefit patients with T1–T2 cN0 early breast cancer and 1–2 positive sentinel lymph nodes (SLN) undergoing breast-conserving surgery (BCT). This paper reports cALND rates in the clinical routine for patients who had higher (T3–T4) tumor stages and/or underwent mastectomy but otherwise met the ACOSOG Z0011 eligibility criteria. Aim of this study is to determine cALND time trends and non-sentinel axillary metastases (NSAM) rates to estimate occult axillary tumor burden.

**Methods:**

Data were included from patients treated in 179 German breast cancer centers between 2008 and 2015. Time-trend rates were analyzed for cALND of patients with T3–T4 tumors separated for BCT and mastectomy and regarding presence of axillary macrometastases or micrometastases.

**Results:**

Data were available for 188,909 patients, of whom 19,009 were identified with 1–2 positive SLN. Those 19,009 patients were separated into 4 cohorts: (1) Patients with T1–T2 tumors receiving BCT (ACOSOG Z0011 eligible; *n* = 13,741), (2) T1–T2 with mastectomy (*n* = 4093), (3) T3–T4 with BCT (*n* = 269), (4) T3–T4 with mastectomy (*n* = 906). Among patients with T3–T4 tumors, cALND rates declined from 2008 to 2015: from 88.2 to 62.6% for patients receiving mastectomy and from 96.6 to 58.1% in patients receiving BCT. Overall rates for any NSAM after cALND for cohorts 1–4 were 33.4%, 42.3%, 46.9%, 58.8%, respectively.

**Conclusions:**

The cALND rates have decreased substantially in routine care in patients with ‘extended’ ACOSOG Z0011 eligibility criteria. Axillary tumor burden is higher in these patients than in the ACOSOG Z0011 trial.

## Introduction

Surgical management of early breast cancer (EBC) comprises margin-free removal of the tumor from the breast and histopathologic determination of axillary lymph node status. The lymph node status is necessary for estimating individual prognosis regarding cancer recurrence in the treatment decision process for adjuvant therapy, i.e. radiotherapy [1] or chemotherapy [2]. So lymph node sampling has a diagnostic value in this context and can be performed either by the selective removal of sentinel lymph node(s) (SLN) or by a systematic axillary lymph node dissection (ALND). The extent of radical surgery to the axilla, and the accompanying morbidity, has been reduced through implementation of axillary staging via SLN dissection (SLND). SLND is the standard procedure for axillary staging in clinically node-negative patients [3]. For patients with affected SLN, a subsequent completing ALND (cALND) had been recommended [4].

The practice-changing results from the American College of Surgeons Oncology Group (ACOSOG) Z0011 trial called into question the practice of cALND [5]. This trial assessed the impact of omitting cALND in clinically node-negative (cN0) EBC patients undergoing breast-conserving therapy (BCT) with tumors size ≤ 5 cm (i.e. T1–T2) and 1 or 2 positive SLN. SLND alone resulted in equivalent loco-regional control, disease-free survival, and overall survival rates in comparison to cALND at 10-year follow-up [6]. Remarkably, the cALND group contained 97 out of 355 patients (27.3%) with additional non-sentinel axillary metastasis (NSAM) in lymph nodes removed by cALND. This implied that unremoved tumor-affected axillary lymph nodes seem not to be prognostically relevant for many patients when receiving guideline-adherent adjuvant treatment. Thus, the identification of occult axillary metastases does not appear to result in improved oncological outcome [7].

However, the ACOSOG Z0011 trial has been criticized for its low statistical power because of slow recruitment and premature termination of the study. Furthermore, mainly low-risk patients were included, which may have influenced survival outcomes. So the applicability of the trial results to routine clinical practice has been questioned [8, 9]. Nevertheless, the idea of omitting cALND in subgroups has been analyzed in further randomized controlled trials (RCTs): first for axillary micrometastases in the IBCSG 23-01 trial [10, 11], but then also in specific radiotherapy settings, such as in the AMAROS trial [12] or the OTOASOR trial [13]. These trials confirmed the results of ACOSOG Z0011, showing no oncological benefit for cALND with even higher rates of NSAM. Comparable NSAM rates were also found in a study on a German cohort that met ACOSOG Z0011 eligibility criteria yet was treated with cALND [14].

The results from the ACOSOG trial have been incorporated into guidelines [15] and the clinical routine [3, 16], but surgeon acceptance still underlies high variability [17]. On the other hand, clinical practice based on the ACOSOG Z0011 results has the advantages of reducing morbidity [18] and healthcare costs [19].

It is the aim to identify further patient populations in which a reduced axillary surgery is possible without comprising oncological outcome. The application of the clinical recommendations based on ACOSOG Z0011 also to patients with higher tumor stages (T3–T4) and to patients undergoing mastectomy are being discussed [20] and to some extent are already being implemented in the clinical routine for SLN-positive patients [21, 22].

In this study we evaluated patients with ‘extended’ ACOSOG Z0011 criteria by analyzing the annual rate of cALND in a large prospectively collected cohort from 179 German breast cancer units (BCU). Furthermore, we compared the rates of NSAM in a cohort of patients who met ACOSOG Z0011 eligibility criteria except for having higher tumor stages and/or mastectomy versus a cohort who did meet all ACOSOG Z0011 eligibility criteria. For the evaluation of NSAM, four cohorts were compared: (1) patients who met all ACOSOG Z0011 eligibility criteria, (2) patients who met all ACSOGO Z0011 eligibility criteria but had mastectomy, (3) patients who met all ACSOGO Z0011 eligibility criteria but had T3–T4 tumors (yet still received BCT), (4) patients who met all ACSOGO Z0011 eligibility criteria but had T3–T4 tumors and mastectomy. Additionally, factors associated with the performance of cALND in T3–T4 cohorts were identified using multivariable logistic regression analyses.

The aim of this study is to present an estimation of occult axillary tumor burden in cohorts who do not met all ACOSOG eligibility criteria to support clinical decision-making.

## Methods

### Database

Data were obtained from a voluntary benchmarking project in Germany. The participating units contributed clinical, surgical, and pathological data from patients with EBC to the West German Breast Center (WBC), Düsseldorf, Germany. All patient treated at one of the participating units are automatically registered in the database. The WBC provides quality control through an annual benchmarking report [23]. The data are also used for the German Cancer Society’s periodical re-certification process to be a certified BCU. Collaborating BCUs collected the data prospectively. Thus, this is a post hoc analysis of registry data.

The validity and quality of the data registered in the WBC tumor documentation system are assessed through a detailed benchmarking system. Comparative quality assessment through benchmarking requires accurate recording of treatment data. The credibility of the tumor documentation is examined for validation purposes. Besides the statistical data-check procedures, in-house data monitoring by clinical research associates is performed twice per year in the participating BCUs.

The study was approved by the ethics committee of the University of Heidelberg and was conducted in accordance with the Declaration of Helsinki. The study was deemed to be without risk, including only analysis of anonymized, routinely collected data; consequently, the ethics committee of the University of Heidelberg did not request approval for patient consent for this designated analysis. Informed consent was obtained from all individual participants for the data acquisition of the benchmarking process.

### Eligibility criteria

For this analysis, anonymized data from all patients with EBC treated between 1 January 2008 and 31 December 2015 were obtained from the WBC database. From this dataset, 4 cohorts of patients were extracted with clinically negative lymph nodes and 1–2 affected SLN: (1) patients with T1–T2 tumors receiving BCT, (2) patients with T1–T2 tumors receiving mastectomy, (3) T3–4 with BCT, (4) T3–4 with mastectomy. The first cohort represents patients who meet all the eligibility criteria of the ACOSOG Z0011 trial. The other three cohorts meet all the eligibility criteria of the ACOSOG Z0011 trial except for having higher tumor stages and/or receiving mastectomy. Adjuvant treatment was performed according to guidelines.

**Fig. 1:**
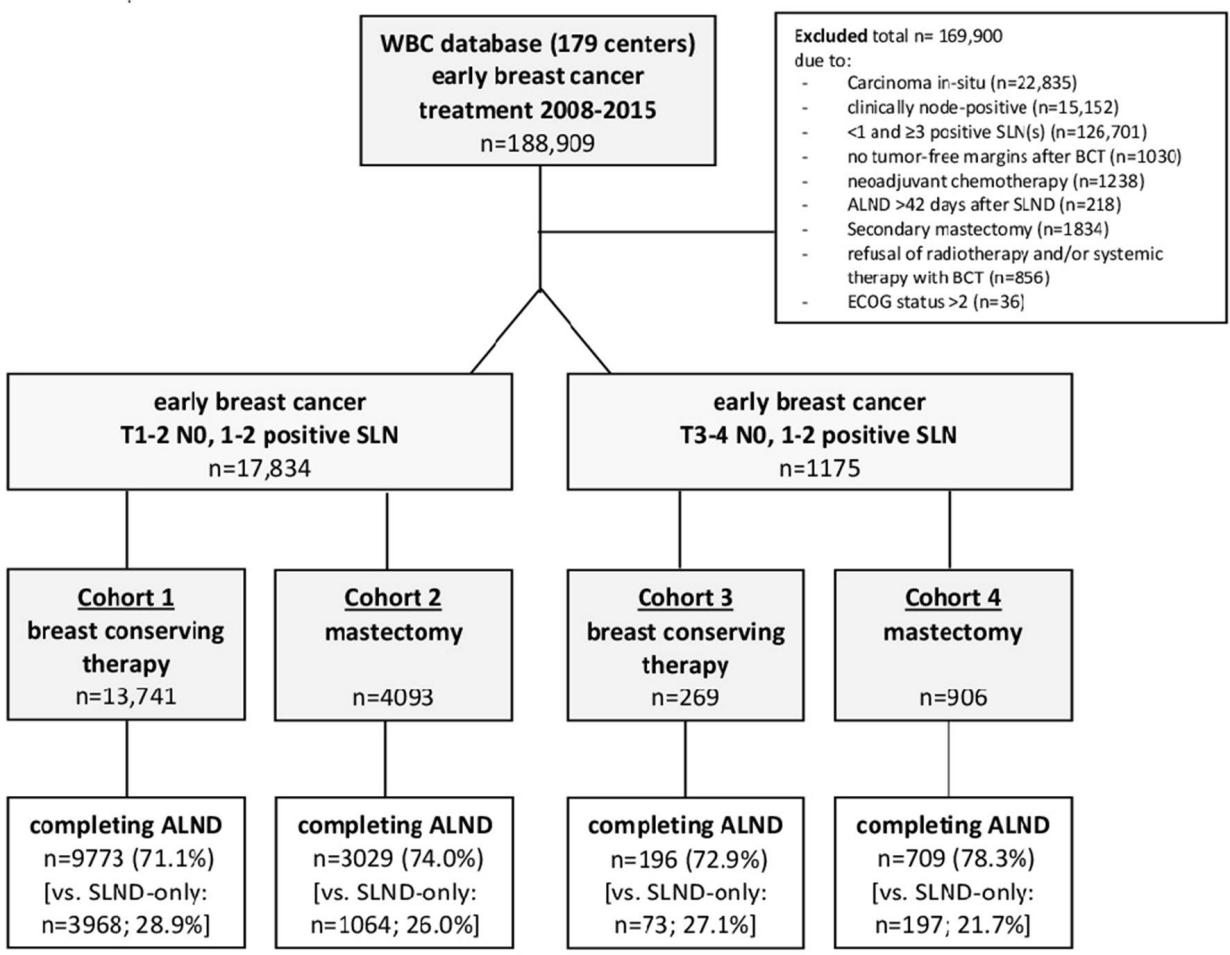
Consort diagram presenting study cohorts

In our study we selected patients for the analysis according to the eligibility criteria of the ACOSOG Z0011 trial by Giuliano et al. shown in Fig. 1. Tumor stages of the patients with the extended ACOSOG Z011 eligible criteria were grouped according to the recent TNM classification including inflammatory breast cancer defined as T4(d). Unlike in the ACOSOG Z0011 trial, all analyses for this study were made using the pathological tumor stage (pT) due to the inconsistent documentation of the clinical tumor (cT) stage in our database. This substitution seems reasonable, given the high concordance between the two types of staging [16]. All participating institutes that provided patient data to the database were labeled before the beginning of the analysis either as university hospitals, academic hospitals (i.e. associated to a university in their function as an academic teaching hospital) or non-academic hospitals. For annual caseload three groups were pre-defined: 100; 101–250; > 250 cases/year.

### Statistical analysis

Patient and tumor characteristics were reported as absolute and relative frequencies. Annual percentages of cALND were calculated and presented as a longitudinal time-trend analysis for the period from 2008 to 2015. This was done for cases with T3–T4 tumors and 1–2 tumor-affected SLN, who were separated for BCT or mastectomy and for presence of sentinel micrometastases or macrometastases. The difference in both variables over the course of time was graphically illustrated and analyzed using logistic regression models with repeated measurements. Multivariable logistic regression was used to identify factors associated with performing cALND in patients with T3–T4 cN0 EBC and 1–2 tumor-affected SLN. These regression analyses were restricted to the subset of patients who were treated since the publication of the ACOSOG Z0011 results (in December 2010). Due to the large sample size in the regression analysis (*n* = 825), p-values of < 0.001 were considered as statistically significant in a descriptive sense. Missing data were not imputed. All statistical analyses were performed with R, version 3.5.1.

## Results

### Study sample

The entire study cohort included 188,909 patients with EBC treated between 2008 and 2015 at 179 BCU in Germany. Of these, 169,900 patients were excluded for the reasons shown in Fig. 1, leaving 19,009 patients with clinically node-negative lymph nodes and 1–2 positive SLN (17,843 with T1–T2 and 1175 with T3–T4). These 19,909 patients were then sorted into 4 cohorts: (1) 13,741 patients who met all eligibility criteria for the ACOSOG Z0011 trial,(2) 4093 patients who had mastectomy for T1–T2 tumors, (3) 269 patients who had BCT for T3–T4 tumors, and (4) 906 patients who had mastectomy for T3–T4 tumors. Then cALND was performed (after histological confirmation of metastases in 1–2 SLN) in 9773 patients (71.1%) in cohort one, 3029 patients (74.0%) in cohort two, 196 patients (72.9%) in cohort three, and 709 patients (78.3%) in cohort four. All other patients received SLND only (Fig. 1).

These four cohorts did not differ substantially from each other: most cases were hormone receptor positive, HER2 negative, and had an intermediate tumor grading. Higher median age and more patients with a lower ECOG performance score were seen in cohorts 2–4 in comparison to the ACOSOG Z0011 eligible cohort (cohort 1). Table 1 presents detailed patient and tumor characteristics.

**Table 1:** Characteristics of the entire study cohort and the cohorts 1–4 (absolute and relative frequencies)

Characteristics	Entire study cohort	*Cohort 1* T1–T2 N0, 1–2 positive SLN and BCT	*Cohort 2* T1–T2 N0, 1–2 positive SLN and mastectomy	*Cohort 3* T3–T4 N0, 1–2 positive SLN and BCT	*Cohort 4* T3–T4 N0, 1–2 positive SLN and mastectomy
*n*	188,909	13,741	4093	269	906
Age					
Median (range)	62 (18–100)	60 (23–95)	68 (21–95)	64 (27–89)	70 (20–97)
Missing	0	0	0	0	0
Age group, *n* (%)					
≤ 50 year	41,127 (21.8)	3307 (24.1)	846 (20.7)	50 (18.6)	144 (15.9)
> 50 year	147,782 (78.2)	10,434 (75.9)	3247 (79.3)	219 (81.4)	762 (84.1)
ECOG status, *n* (%)					
0	141,063 (83.8)	11,093 (89.4)	2735 (77.1)	197 (82.8)	565 (71.7)
1	22,275 (13.2)	1219 (9.8)	646 (18.2)	35 (14.7)	171 (21.7)
2	4261 (2.5)	96 (0.8)	168 (4.7)	6 (2.5)	52 (6.6)
≥ 3	697 (0.4)	–	–	–	–
Missing	20,633	1333	544	31	118
T stage, *n* (%*)					
T0	3548 (1.9)	–	–	–	–
Tis	21,656 (11.5)	–	–	–	–
T1	93,389 (49.5)	7998 (58.2)	1242 (30.4)	–	–
T2	56,463 (29.9)	5740 (41.8)	2849 (69.6)	–	–
T3	8144 (4.3)	–	–	175 (65.1)	627 (69.2)
T4	5463 (2.9)	–	–	94 (34.9)	279 (30.8)
Missing	246	0	2	0	0
ER status, *n* (%*)					
Positive	156,248 (83.4)	12,455 (90.7)	3710 (90.7)	246 (91.5)	811 (89.6)
Negative	31,072 (16.6)	1277 (9.3)	382 (9.3)	23 (8.6)	94 (10.4)
Missing	1589	9	1	0	1
PR status, *n* (%*)					
Positive	135,903 (72.6)	11,177 (81.4)	3226 (78.9)	221 (82.16)	692 (76.46)
Negative	51,372 (27.4)	2555 (18.6)	864 (21.1)	48 (17.84)	213 (23.54)
Missing	1634	9	3	0	1
HER2 status *n* (%*)					
Positive	22,573 (13.1)	1346 (9.9)	516 (12.8)	27 (10.1)	99 (11.1)
Negative	149,374 (86.9)	12,199 (90.1)	3516 (87.2)	241 (89.9)	793 (88.9)
Missing	16,962	196	61	1	14
Lymphovascular invasion, *n* (%*)					
Yes	36,769 (22.2)	5423 (42.7)	1752 (46.3)	137 (54.4)	484 (57.1)
No	128,851 (77.8)	7277 (57.3)	2030 (53.7)	115 (45.6)	363 (42.9)
Missing	23,289	1041	311	17	59
Grading, *n* (%*)					
G1 (low)	27,019 (14.6)	1841 (13.4)	329 (8.1)	15 (5.6)	57 (6.3)
G2 (intermediate)	104,665 (56.7)	8793 (64.1)	2709 (66.3)	186 (69.1)	616 (68.0)
G3 (high)	52,853 (28.6)	3089 (22.5)	1051 (25.7)	68 (25.3)	233 (25.7)

*T* tumor stage, *ECOG* Eastern Cooperative Oncology Group, *ER* estrogen, *PR* progesterone

*The missing values were not included in the calculation of the relative frequencies

### cALND Time-Trend Analyses for T3–T4 cN0 and 1–2 positive SLN

**Fig. 2:**
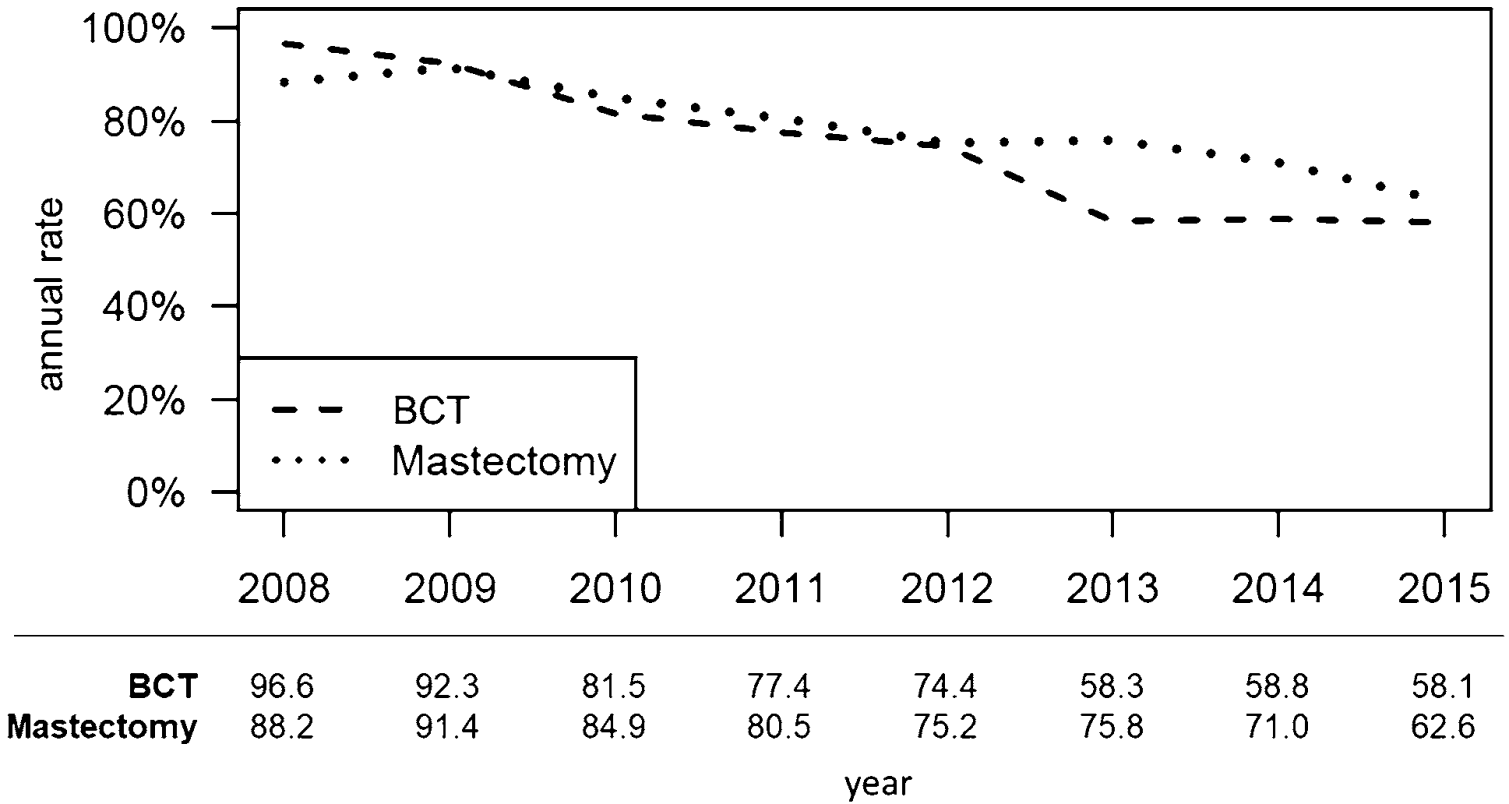
Completing axillary lymph node dissection rate in patients with T3–T4 N0 early breast cancer and one or two tumor-affected sentinel lymph nodes separated for breast-conserving therapy or mastectomy between 2008 and 2015 (BCT: breast-conserving therapy)

**Fig. 3:**
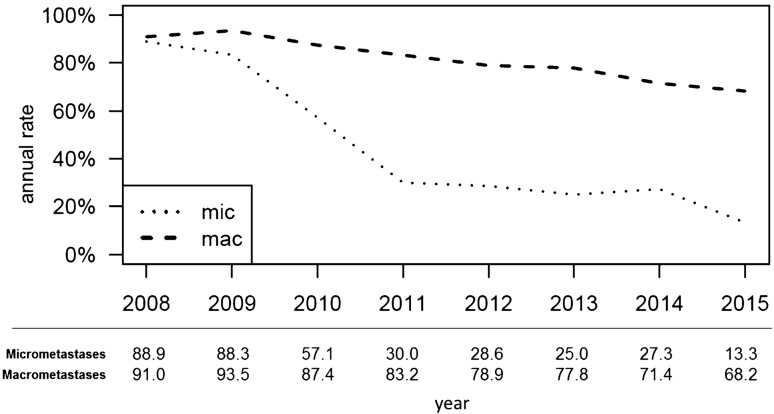
Rate of completing axillary lymph node dissection in patients with T3–T4 N0 early breast cancer and one or two tumor-affected sentinel lymph nodes, separated for sentinel micrometastases (mic) or macrometastases (mac), between 2008 and 2015

The annual cALND rate declined from 96.6% (in 2008) to 58.1% (in 2015) for patients receiving BCT, and from 88.2% (in 2008) to 62.6% (in 2015) for patients receiving mastectomy (time trend *p* < 0.001; curve separation: *p* = 0.164; Fig. 2). The cALND rate declined from 88.9 (in 2008) to 13.3% (in 2015) for patients with micrometastases (total 54/121 cases), and from 91.0 (in 2008) to 68.2% (in 2015) for patients with macrometastases (total 851/1054 cases; time trend *p* < 0.001, curve separation *p* = 0.009; Fig. 3).

### Factors associated with performance of cALND

In multivariable analyses, higher annual hospital case load, younger age, macrometastases, performing a mastectomy and lower number of SLN removed as well as higher number of affected SLN had significant influences on the performance of cALND in patients with T3–T4 cN0 EBC and 1–2 tumor-affected SLN. Immunohistochemical parameters such as positivity for hormone or HER2 receptor and hospital type affiliation were not significant factors (Table 2).

**Table 2:** Multivariable analysis of factors influencing the decision to perform completing axillary lymph node dissection in patients with T3–T4 cN0 early breast cancer and one or two tumor-affected sentinel lymph nodes since publication of the ACOSOG Z0011 results

Variable	Odds ratio (95% CI)	*p* value
Type of hospital		
General, non-academic hospital	Reference	
Academic teaching hospital	1.12 (0.75–1.67)	0.576
University hospital	1.12 (0.50–2.67)	0.783
Annual hospital caseload		
< 150	Reference	
150–249	1.53 (0.97–2.43)	0.07
≥ 250	2.16 (1.30–3.62)	0.003
Age (years)	0.96 (0.94–0.98)	< 0.001
Tumor stage		
3	Reference	
4	0.99 (0.63–1.56)	0.97
Type of metastasis		
Macro	Reference	
Micro	0.08 (0.04–0.17)	< 0.001
Sentinel removed	0.70 (0.62–0.80)	< 0.001
Sentinel affected	2.08 (1.31–3.37)	0.002
Surgical procedure		
BCT	Reference	
BCT with re-excision	0.92 (0.39–2.25)	0.84
Mastectomy	1.58 (0.94–2.64)	0.08
Mastectomy with re-excision	1.78 (0.25–36.9)	0.62
Grading		
G1 (low)	Reference	
G2 (intermediate)	1.05 (0.47–2.22)	0.90
G3 (high)	1.21 (0.50–2.84)	0.67
Lymphovascular invasion		
No	Reference	
Yes	1.28 (0.86–1.91)	0.23
Histological subtype		
Ductal	Reference	
Lobular	0.61 (0.71–1.79)	0.61
Other	0.33 (0.59–5.94)	0.33
Hormone receptor status		
Negative	Reference	
Positive	0.89 (0.37–1.98)	0.78
HER2 receptor status		
Negative	Reference	
Positive	1.12 (0.59–2.18)	0.74
ECOG performance status		
0	Reference	
1	0.58 (0.36–0.93)	0.02
2	1.12 (0.48–2.69)	0.81

*CI* confidence interval, *BCT* breast-conserving therapy, *ECOG* Eastern Cooperative Oncology Group

### Non-sentinel axillary metastases in the cALND cohorts

**Fig. 4:**
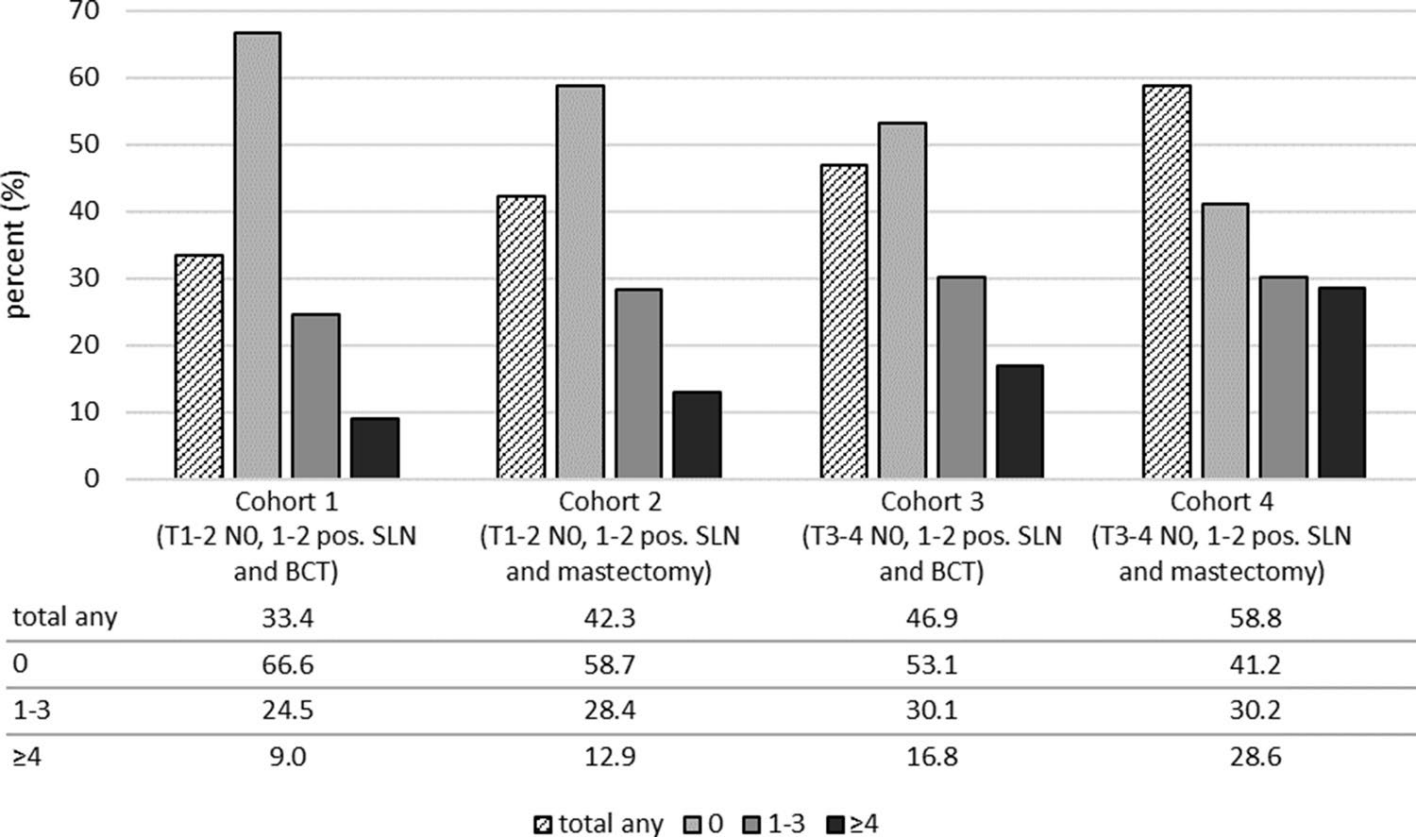
Rates for non-sentinel metastases after completing axillary lymph node dissection for cohorts 1–4, presented as total any and separated into three subgroups (0, 1–3 and ≥ 4 NSAM) (*SLN* sentinel lymph node, *BCT* breast-conserving therapy)

In the cALND of our patients eligible for ACOSOG Z0011 (cohort 1), 3264 out of 9773 cases (33.4%) were found to have additional NSAM in the axillary lymph node dissection specimens. This rate was higher in the other cohorts: 42.3% in cohort 2, 46.9% in cohort 3, and 58.8% in cohort 4. The portion of patients with 4 or more NSAM in the axillary specimen was: 9.0% in cohort one, 12.9% in cohort two, 16.8% in cohort three, and 28.6% in cohort four (Fig. 4).

## Discussion

Since the publication of the ACOSOG Z0011 trial, an increasing number of patients have been treated accordingly [3, 17, 22, 24]. Here we compared cALND rates in patients who did versus did not fulfill all eligibility criteria of the ACOSOG Z0011 trial. We found decreasing rates of cALND over time in patients with T3–4 cN0 EBC with 1–2 tumor-involved SLN undergoing BCT or mastectomy. There are already reports from clinical routine cohorts that cALND is omitted also in SLN-positive patients outside the ACOSOG Z0011 criteria, as already shown for the same cohort in patients with mastectomy [21] or for a Dutch cohort with higher tumor stage [22]. Kenny et al. have suggested that the ACOSOG Z0011 trial significantly altered axillary management in all EBC patients with positive SLN, not only in those receiving BCT [25]. Interestingly, Yao et al. reported from the US National Cancer Data Base that even in the pre-Z0011 era (1998–2011), around 22% of mastectomy patients who were otherwise ACOSOG Z0011 eligible did not undergo cALND [26].

It must be emphasized that until now no randomized trial has been published to support a less extensive axillary surgery approach in T3–T4 cN0 SLN-positive EBC cases or in patients undergoing mastectomy. The prospective, randomized trials, AMAROS and OTOASOR, analyzed the omission of cALND in comparison to axillary radiotherapy in patients with EBC and clinically node-negative lymph nodes with positive SLN. Both trials included patients with mastectomy to a small extent: 17% (248/1425 in the intervention arm) in AMAROS and 16% (47/474 in the intervention arm) in OTOASOR. Nonetheless, these sample sizes were underpowered for a subgroup analysis [12, 13]. Another important aspect is the effect of radiotherapy on oncological outcome. The results of the ACOSOG Z0011 [6] and IBCSG 23-01 [10] trials cannot be extrapolated to SLN-positive patients treated with mastectomy, as these patients do not routinely receive adjuvant radiation therapy according to recent guidelines, e.g. the German S3 guideline [27]. Nonetheless, studies suggest that radiotherapy can be as effective as ALND in patients with mastectomy and nodal-positive disease for overall and recurrence-free survival rates [28].

Currently, randomized trials are being prepared to deliver high evidence for node-positive patients undergoing mastectomy. For example, the randomized controlled BOOG 2013–07 trial aims to investigate whether cALND can be safely omitted in SLN-positive EBC patients treated with mastectomy [29]. Further, the POSNOC trial, with a planned sample size of 1900 and a stratification for mastectomy, will assess whether SLND-alone is non-inferior to axillary radiation for women with ≤ 2 macrometastases [30]. The SENOMAC trial includes patients with T1–T3 tumors and patients undergoing either BCT or mastectomy, and it is investigating whether SLND-alone is sufficient as axillary staging [31].

The reasons why cALND is sometimes omitted despite the lack of evidence to support omission have not been systemically examined, but it can be assumed this affects mainly vulnerable subgroups with reduced general health conditions, e.g. due to higher age. A retrospective study from the Netherlands has shown that the omission of complete axillary staging was common in selected elderly EBC patients receiving endocrine therapy with ≥ 2 comorbidities and had no apparent impact on regional control and 10-year overall survival [32]. Similar result have been shown earlier by analyses from the US SEER database [33]. Also, in our multivariable analysis with T3–T4 EBC, higher patient age was a significant influencing factor in the omission of cALND (Tab. 2). This is comparable to earlier analyses [22]. Further important factor influencing treatment decisions to perform a cALND was the nodal tumor burden, determined by the number of involved SLNs and the number of removed SLNs. In routine clinical practice, physicians rather omitted cALND in patients with fewer tumor-affected SLNs and more removed SLNs, suggesting a higher likelihood of no further non-sentinel metastasis. Moreover, institutional factors like treatment in centers with a high annual case volume lead to higher probability of cALND which might be explained by standardized treatment procedures in high volume settings.

Results from trials were extrapolated when implementing them in clinical routine. This is based on clinically analogous assumptions in the routine decision-making process. Since larger tumors have a higher likelihood of axillary lymph node involvement, the early studies on the implementation of the SLND technique 20 years ago mostly restricted their inclusion criteria to EBC patients with tumor size ≤ 5 cm [34, 35]. Thus, in clinical routine the use of SLND was also extrapolated for larger tumors and multicentric disease. A comparable phenomenon can also be observed after the results from the ACOSOG Z0011 trials were published. Nonetheless, application of the conclusions from trial results to other populations of patients beyond the inclusion criteria must be considered with caution due to the lack of evidence and the possibility of deteriorating the oncological outcome.

In the cALND arm of the ACOSOG Z0011 trial, 27.3% of the patients had additional involved NSAM and 13.7% even had ≥ 4 NSAM [10]. Higher rates of NSAM in the control groups with cALND have been reported in the other randomized trials that compared the omission of cALND with radiotherapeutic interventions. In the AMAROS trial, the portion of patients with NSAM was 32.7%, and the portion of patients with ≥ 4 NSAM was 8.3% [12]. In the OTOSAR trial, the portion of patients with NSAM was 38.5% [13]. In our study, the portion of patients with NSAM was 33.4% after cALND in the ACOSOG-eligible cohort, which is slightly higher than in the ACOSOG Z0011 trial. In contrast, the portion of patients with ≥ 4 NSAM was lower in our study (9.0%) than in the ACOSOG Z0011 trial [14]. Some studies have found that clinicopathological factors (such as lobular type or multifocality), type of SN metastases, and the number SN with macrometastases might possibly be related to NSAM rates and could be used in the treatment decision process [36].

Nonetheless, there have not been any studies so far that have reported the portion of patients with NSAM in cohorts not meeting the ACOSOG eligibility criteria, although nomograms have also been established for patients with mastectomy [37]. In our cohorts not meeting the ACOSOG eligibility criteria, higher portions of patients with NSAM involvement were found in comparison to the ACOSOG-eligible cohort. These were: 41.3% in T1–T2 with mastectomy (cohort 2), 46.9% for T3–T4 with BCT (cohort 3), and 58.8% for T3–T4 with mastectomy (cohort 4). Correspondingly, the portion of patients with ≥ 4 NSAM found in the cALND specimen range from 12.9 to 28.6%, which suggests a high residual tumor burden if cALND is omitted (Fig. 4).

Axillary lymph node status still has an important influence on adjuvant treatment decisions, especially for patients with favorable tumor biology, as previous discussed for this cohort [14]. Retrospective cohorts from the Netherlands have illustrated this impact on the decision for adjuvant chemotherapy if cALND was omitted: T1–T2 N0 SLN-positive EBC patients treated with cALND had a higher probability of receiving adjuvant chemotherapy compared with SLND-only patients [38]. The authors emphasized that treatment decisions were not only based on guidelines and tumor characteristics but also on the preferences of physicians and patients. Further studies will have to identify factors that prognosticate good outcomes even without cALND, as was done for example for patients with microscopic extracapsular extension [39].

The use neoadjuvant chemotherapy, which was excluded from ACOSOG Z011, contributes to the goal of minimizing the use of ALND and its associated morbidity. Patients with larger tumors and an aggressive subtype, i.e. HER2-enriched and TN will receive neoadjuvant chemotherapy (NAC). In these patients SLNB after NAC in clinically node-negative patients has the potential to downstage microscopic nodal disease and thus avoid ALND [40].

This study is limited by its post hoc nature and lack of outcome data. Furthermore, incomplete information about the cT stage led to the use of the pT stage to determine whether patients met the ACOSOG Z0011 inclusion criteria. We considered this approach reasonable though, because the pT stage and cT stage were concordant for most cases in our cohort.

## Conclusion

To reduce the morbidity in the management of breast cancer patients, surgeons should aim to minimize the rates of cALND among clinically node-negative patients with pathologically node-positive disease. Rates for cALND have decreased substantially in routine care. A rising prevalence of additional NSAM tumor burden is linked with the extension of the ACOSOG Z0011 study eligibility criteria. The goal is to identify further patient populations in which a reduced axillary surgery is possible without compromising oncological outcome. So far, there is no evidence that the substantial tumor burden left behind has no disadvantage on outcome in cases with extended ACOSOG Z0011 eligibility criteria, especially T3–T4 tumors. However, selected subgroups might still benefit in saving them from unnecessary morbidity from cALND in an individual risk–benefit analysis.

## Data Availability

The datasets are available from the corresponding author on reasonable request.
